# Immersive virtual reality (VR) training increases the self-efficacy of in-hospital healthcare providers and patient families regarding tracheostomy-related knowledge and care skills: A prospective pre–post study

**DOI:** 10.1097/MD.0000000000028570

**Published:** 2022-01-14

**Authors:** Dung-Hung Chiang, Chia-Chang Huang, Shu-Chuan Cheng, Jui-Chun Cheng, Cheng-Hsien Wu, Shiau-Shian Huang, Ying-Ying Yang, Ling-Yu Yang, Shou-Yen Kao, Chen-Huan Chen, Boaz Shulruf, Fa-Yauh Lee

**Affiliations:** a Department of Critical Care Medicine, Taipei Veterans General Hospital, Taipei, Taiwan; b Faculty of Medicine, Taipei, Taiwan; c National Yang-Ming Chiao Tung University, Taipei, Taiwan; d Department of Medical Education, Taipei Veterans General Hospital, Taipei, Taiwan; e Medical Innovation Research Office, Clinical Innovation Center, Department of Medical Education, Taipei Veterans General Hospital, Taipei, Taiwan; f Division of Respiratory Therapy, Department of Chest Medicine, Taipei Veterans General Hospital, Taipei, Taiwan; g Division of Family Dentistry, Department of Stomatology, Taipei Veterans General Hospital, Taipei, Taiwan; h New South Wales Sydney University, Australia.

**Keywords:** familiarity, knowledge, satisfaction, self-efficacy, tracheostomy, virtual reality

## Abstract

**Background:**

Virtual reality (VR)-based simulation in hospital settings facilitates the acquisition of skills without compromising patient safety. Despite regular text-based training, a baseline survey of randomly selected healthcare providers revealed deficiencies in their *knowledge*, *confidence*, *comfort*, and care skills regarding tracheostomy. This prospective pre–post study compared the effectiveness of *regular* text- and VR-based *intervention* modules in training healthcare providers*’* self-efficacy in tracheostomy care skills.

**Methods:**

Between January 2018 and January 2020, 60 healthcare providers, including physicians, nurses, and respiratory therapists, were enrolled. For the intervention, a newly developed head-mounted display (HMD) and web VR materials were implemented in training and clinical services. Subsequently, in-hospital healthcare providers were trained using either text or head-mounted display virtual reality (HMD-VR) materials in the *regular* and *intervention* modules, respectively. For tracheostomy care skills, preceptors directly audited the performance of trainees and provided feedback.

**Results:**

At baseline, the degree of trainees*’* agreement with the self-efficacy-related statements, including the aspects of *familiarity, confidence*, and *anxiety* about tracheostomy-related knowledge and care skills, were not different between the control and *intervention* groups. At follow-up stage, compared with the *regular* group, a higher percentage of *intervention* group*’* trainees reported that they are “strongly agree” or “somewhat agree” that the HMD-VR simulation increases their self-efficacy, including the aspects of *familiarity* and *confidence*, and reduced their *anxiety* about tracheostomy-related knowledge and care skills. After implementation, a higher degree of trainees*’* average satisfaction with VR-based training and VR materials was observed in the *intervention* group than in the *regular* group. Most reported that VR materials enabled accurate messaging and decreased anxiety. The increasing trend of the average written test and hands-on tracheostomy care skills scores among the *intervention* group trainees was significant compared to those in the *regular* group. The benefits of HMD-VR simulations and web-VR material-based clinical services for in-hospital healthcare providers and patient families persisted until 3 to 4 weeks later.

**Conclusion:**

The current study suggests that VR materials significantly enhance trainees’ self-efficacy (increased familiarity, increased confidence, and reduced anxiety) and their satisfaction with the training, while motivating them to use acquired knowledge and skills in clinical practice.

## 1 Introduction

The number of patients requiring prolonged mechanical ventilation (MV) after an acute illness or injury is steadily increasing worldwide.^[1,2]^ For ventilator-dependent or difficult-to-wean patients, tracheostomy has several advantages over an endotracheal tube (ETT). For example, conversion from ETT intubation to tracheostomy has been found to significantly improve weaning parameters in difficult-to-wean patients who were subsequently weaned off MV successfully.^[3]^ Compared with an ETT, a tracheostomy is more beneficial for ventilator-dependent or difficult-to-wean patients, such as offering more efficient suction, enabling earlier communication, and making oral feeding possible.^[4]^ Additionally, tracheostomy increases the possibility of transferring ventilator-dependent patients from the intensive care unit (ICU) to general wards.^[5,6]^ The time required to perform tracheostomy is individualized and based on daily weaning assessment after an initial ETT. It has been reported that tracheostomy 21 days after ETT is associated with a higher rate of failure to wean from MV, longer ICU stay, and higher ICU mortality.^[7]^ Recent studies have reported that tracheostomy can be suggested for ventilator-dependent or difficult-to-wean acute respiratory failure patients 14 days after emergent ETT.^[8]^

In Taiwan, difficult-to-wean patients are usually transferred to respiratory recovery centers (RCCs) for weaning. In these patients, tracheostomy before transfer to the RCC was associated with a significantly shorter MV duration, higher weaning rate, and shorter length of hospital stay.^[9,10]^ Another similar study from Taiwan showed that in-hospital mortality and ventilator-associated pneumonia significantly decreased in the pre-RCC tracheostomy group compared with the control group.^[10]^ Nevertheless, a significant proportion of difficult-to-wean patients or families hesitate to provide consent for tracheostomy because of insufficient information received from healthcare providers. Recent studies from Taiwan reported that tracheostomy in critically ill patients is often delayed for more than 14 days, with the mean time to tracheostomy being between 18.5 and 36.7 days.^[11,12]^

ETT or elective tracheostomy is recommended to prepare for re-exploration, induce anesthesia faster, and avoid airway compromise from trauma-related postoperative airway edema or postsurgery anatomical changes in the head and neck area among patients with oropharyngeal cancer.^[5,13–19]^ Before undergoing major surgery, elective tracheostomy in oropharyngeal cancer patients can reduce pneumonia, decrease postoperative delirium, and shorten the duration of MV dependence and ICU stay.^[20]^

With advances in intensive care technology, the number of patients with tracheostomy tubes discharged to long-term care facilities is increasing.^[8,21,22]^ Good tracheostomy care requires regular suction, stoma care, nutrition, speech therapy, and periodic changes to the tracheostomy tube.^[23]^ Studies have shown that tracheostomy patients are often at risk of suboptimal care and increased morbidity owing to insufficient skills and experience of healthcare providers.^[24,25]^

Recent research has shown a lack of familiarity and confidence in tracheostomy care among physicians and ward nurses, leading to inevitable and potentially serious complications.^[26,27]^ Hence, it is necessary to design an education program to improve tracheostomy-related knowledge and care skills of in-hospital healthcare providers.^[28–31]^ Virtual reality (VR) simulations provide a controlled environment in which learners can navigate, manipulate, and interact with virtual objects and observe their effects in real time.^[32]^ A recent study reported that game-based VR materials were effective in teaching suction skills for tracheostomies.^[33]^ Nevertheless, that study did not compare the effects of text-based training with those of VR-based training among healthcare providers.

We propose that our newly developed comprehensive VR-based materials and training will enhance the effectiveness of trainees’ learning and application. Overall, our study aimed to compare the effects of text-based materials/training and new VR-based educational materials/training on the self-efficacy of in-hospital healthcare providers with respect to their tracheostomy-related knowledge and care skills and on their satisfaction with the training and materials. Additionally, the degree of trainees’ application of web VR or text in educating patients’ families and the perceptions of patients and families regarding the new VR materials were evaluated through reports from trained in-hospital healthcare providers.

## 2 Methods

### 2.1 Background for the implementation of a new interventional program

To ensure care quality, the education committee in our hospital regularly arranges text-based training to enhance tracheostomy-related knowledge and care skills of healthcare providers. In the *regular* training sessions, the detailed indication for, purpose of, and care skills for tracheostomy were included in the text-based materials for education and clinical services. However, in 2017 to 2018, a random survey of 28 healthcare providers revealed that they lacked *familiarity*, *confidence*, and *comfort* in practice regarding tracheostomy-related knowledge and care skills. Accordingly, the education committee developed head-mounted display (HMD)-VR and web-based VR materials that can be accessed with a desktop, tablet computer, or smartphone. Head-mounted display virtual reality (HMD-VR) materials can be used for group training, whereas web-based VR materials are meant for education outside hospitals or environments where head-mounted display virtual reality facilities cannot be used. In addition to education, program directors hoped that these smartphone-driven web VR materials could be introduced to their patients’ families by in-hospital healthcare providers.

### 2.2 Study design and settings

This prospective, comparative, and pre–post study was conducted in a 2800-bed, 6000-staff medical center and teaching hospital in Taiwan. Our team has developed HMD-VR and smartphone-driven web-VR materials, with the *development* and *implementation* phases taking 2 years. The content of our VR materials was based on text-based materials on tracheostomy-related knowledge and care skills used in *regular* training. The education committee implemented VR materials for education and clinical services. Tracheostomy meetings were conducted regularly to facilitate interactions among critical respiratory care physicians, nurses, and respiratory therapists. During these meetings, in-hospital healthcare providers discussed the practical problems they faced while caring for tracheostomy patients and educating patients’ families.

### 2.3 Contents of VR materials of tracheostomy-related knowledge and care skills for education and clinical services

**Figure 1:**
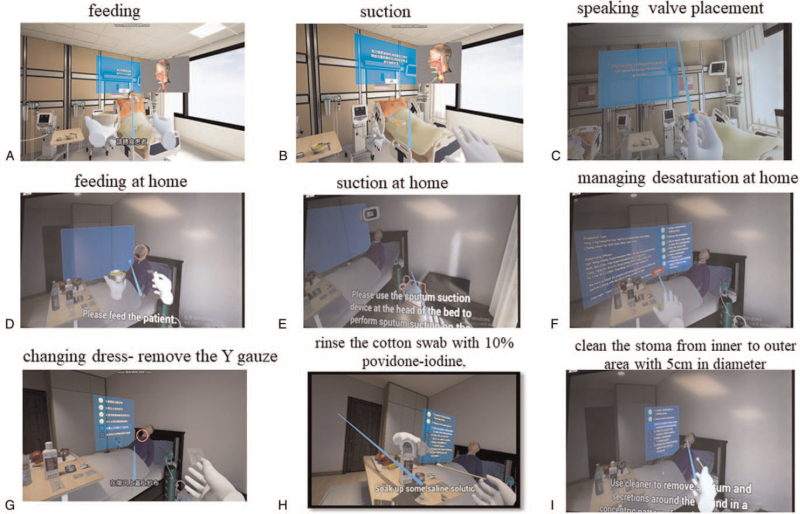
Representative images of our self-developed VR education and service system to demonstrate the differences between patients in the intensive care unit who have undergone endotracheal intubation and tracheostomy in terms of (**A**) feeding, (**B**) suction, (**C**) speaking; demonstrating the care skills including (**D**) feeding at home, (**E**) suction at home, (**F**) managing desaturation at home; showing the correct sequence of changing dress of stoma including (**G**) remove the Y gauze, (**H**) rinse the cotton swab with 10% povidone–iodine, (**I**) clean the stoma from inner to outer area with 5 cm in diameter. Source: Professor Ying-Ying Yang. The corresponding author, Dr Ying-Ying Yang, is the owner of the photos in Figure 1. VR = virtual reality.

Similar to text-based materials used for regular training in *regular* groups, our newly developed VR materials include 2 parts for information collection. In the first part, users can experience the difference between ETT and tracheostomy in aspects such as feeding, suction, and speaking valve placement, and examine the tracheostomy stoma to check for any discharge, infection, bleeding, or obstruction using HMD-VR or smartphone-driven web-VR materials in an ICU setting (Fig. 1). In the second part, in a VR home environment, users can hear suffocation noise due to inappropriate feeding, hear clear breathing sounds after effective suction, listen to virtual patients speaking through the speaking valve, dress the stoma based on step-by-step instructions, and handle emergencies such as aspiration, desaturation, tube dislodgement, and tube occlusion.

### 2.4 Training protocol for the regular group

**Figure 2:**
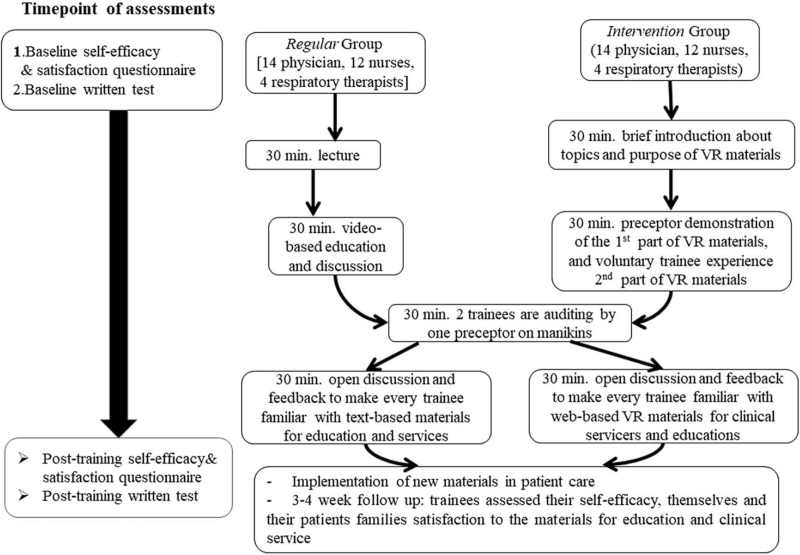
Flow chart of the training between *regular* and *intervention* groups. VR = virtual reality.

A group course facilitated by to 2 to 3 senior instructors was arranged for every 4 to 6 trainees (Table 1). Trainees in the *regular* group (Table 1 and Fig. 2) had 2-hour sessions, beginning with a self-assessed written test on tracheostomy-related knowledge and care skills at baseline (Table 2), followed by a self-assessed questionnaire on self-efficacy and satisfaction at baseline (Table 3) and lectures (30 minutes), video-based education (30 minutes), checklist-based auditing of tracheostomy stoma care skills (30 minutes; using Table 4 and Fig. 2), and open discussion and feedback (30 minutes). The trainees then completed post-training written tests and post-training questionnaires on self-efficacy and satisfaction. Thus, every trainee became familiar with text-based materials for use in clinical services. The answers to the written test were provided after the trainees completed their baseline, post-training, and follow-up self-assessments (Fig. 2). The content of the lecture focused on theoretical knowledge about tracheostomy, including securing the location of a tracheostomy tube, and suctioning and feeding technique; identifying stoma infections; and recognizing tube blockages using a speech valve, and managing complications such as a dislodged tube, accidental decannulation, desaturation, bleeding from the stoma site, or difficulty in changing the tube.

**Table 1:** Basal characteristics between groups.

	Regular group	Intervention group
Case number	30 (14 physicians, 12 nurses vs 4 respiratory therapists)	30 (14 physicians, 12 nurses vs 4 respiratory therapists)
Age range	23–30 yr	21–30 yr
Mean age	27 yr	26 yr
Female/male (no.)	22/8	26 yr
Percentage of distribution of trainees with and without experience of previous training for knowledge and care skills of tracheostomy (yes/no)	20/10	19/11
Percentage of trainees whose prior training reported that they “strongly agree” or “somewhat agree” with the statement of “*prior training met your need with respect to tracheostomy-related knowledge and care skills*”	55%	53%
Percentage of trainees whose prior training reported that they “strongly agree” or “somewhat agree” with the statement of “*prior training provided accurate messages about the tracheotomy-related knowledge and care skills*”	50%	53%
Percentage of trainees reported that they “strongly agree” or “somewhat agree” with the statement of “*you are happy to currently receive training on tracheostomy-related knowledge and care skills*”	76%	79%

**Table 2:** Multiple choice questions (MCQ) written test with single or multiple choices for tracheostomy-related knowledge and care skills for trainees’ self-assessment.

1. Which of the following statement is incorrect about the indication for tracheostomy? **[A]** difficult-to-wean more than 2–3 wk after endotracheal intubation (ETT) of acute respiratory failure patients, [**B**] prolonged mechanical ventilator dependent, [**C**] oropharyngeal cancer patients receiving excision and reconstructive surgery, [**D**] difficult intubation in patients with maxillofacial injury, [**E**] acute airway obstruction caused by foreign bodies in the respiratory tract.
2. Which of the following statement is incorrect about the comparison between prolonged ETT and tracheostomy? **[A]** prolonged ETT will damage vocal cord and induce oral ulcer, **[B]** patients with tracheostomy can speak after training, **[C]** patients with tracheostomy can feed through nasogastric tube, **[D]** patients with prolonged ETT carry higher risk of infection and ventilator-dependent pneumonia, [**E]** patients with tracheostomy can suction and oxygenation more efficiently.
3. Which of the following statement is correct about the care of tracheostomy stoma? [**A**] not need to use any sterile solution, [**B**] clean with normal saline, [**C**] clean with normal saline plus tincture of iodine, [**D**] clean with normal saline plus 10% povidone–iodine, [**E**] check the location and fitness of tracheal tube.
4. Which of the following statement is incorrect about the changing dress of tracheostomy? [**A**] changing dress when there is secretion, [**B**] give 100% oxygen and suction before changing dress, [**C]** raise the head of bed up to 30°–40° before feeding, [**D**] the range of cleaning need to include stoma site and it peripheral and tube holder, [**E**] clean the 10% povidone–iodine with cotton swab to avoid irritation of skin and pigmentation.
5. Which of the following statement is correct about the daily care of tracheostomy stoma? [**A**] the tie of tube should be 1–2 fingers tightness, [**B**] check whether there are signs of infection such as redness, edema, stench discharge of stoma each time and seek medical if it appears, [**C**] tie of tube did not need to change, [**D**] change the tracheal tube each month, [**E**] using the Y gauze can avoid tube and sputum-related skin irritation.
6. Which of the following statement is incorrect about acute management of dislodgement of tracheal tube? [**A**] see patient to hospital without doing anything, [**B**] put back the dislodged tracheal tube immediately, [**C**] open the orifice of stoma with tweezers, [**D**] insert the suction tube to maintain patency of airway temporarily.
7. Which of the correct sequence of changing dress of tracheostomy? 1. remove Y gauze, 2. loosen the tie of tracheostomy, 3. clean first with 10% povidone–iodine and follow by normal saline, 4. clean the peripheral area of stoma with normal saline, 5. cover with aseptic Y gauze. [**A**] 1 + 2 + 3 + 4 + 5, [**B**] 1 + 4 + 3 + 2 + 5, [**C**] 1 + 3 + 4 + 2 + 5, [**D**] 2 + 1 + 4 + 3 + 5

For every MCQ, trainee get 1 point if they choice correct answer or they will get zero point if their answer is wrong. So, the range of score of written test will be 0–7 for any trainee. Answer: 1. [**E**]; 2. [**C**]; 3. [**D** **+** **E**], 4. [**C**]; 5. [**C**]; 6. [**A**]; 7. [**D**].

**Table 3:** Questionnaire for trainees’ self-assessed self-efficacy and satisfaction.

**Baseline** self-efficacy:
1. You are *familiar with* tracheostomy-related knowledge and care skills.
2. You have *confidence* in tracheostomy-related knowledge and care skills.
3. You are not *anxious* about the tracheostomy-related knowledge and care skills.
**Baseline** satisfaction with the prior training:
1. Prior training that you had already meets your needs concerning tracheostomy-related knowledge and care skills.
2. Prior training that you had already provided accurate messages about tracheostomy-related knowledge and care skills.
3. You are happy to receive current training about tracheostomy-related knowledge and care skills.
**Post-training** self-efficacy:
1. You are *familiar with* tracheostomy-related knowledge and care skills.
2. You have *confidence* in tracheostomy-related knowledge and care skills.
3. You are not *anxious* about the tracheostomy-related knowledge and care skills.
**Post-training** satisfaction with the current training:
1. The current training meets your needs concerning tracheostomy-related knowledge and care skills.
2. The current training provides accurate messages about tracheostomy-related knowledge and care skills.
3. You are happy to receive the training about the tracheostomy-related knowledge and care skills.
**Post-training** satisfaction with text-based or smartphone-based VR training and service materials:
1. These materials increase the training effectiveness.
2. These materials achieve the purposes of a paperless environment.
3. These materials will increase the efficacy of clinical serves and benefit your patients and families.
4. You are willing to use the knowledge and skills acquired from these materials in clinical practice.
5. You are willing to recommend these materials to my patients’ families and patients who have undergone or preparing for tracheostomy.

All questions are responded by the degree of agreement to the question [strongly agree (75%–100%), somewhat agree (50%–75%), not somewhat agree (25%–50%), not strongly agree (0%–25%), not applicable]. VR = virtual reality.

**Table 4:** Checklist for evaluating the tracheostomy-related care skills of trainees.

1. Washing hands before caring of tracheostomy.
2. Put on glove and remove Y gauze.
3. Using cotton swab cleaning the stoma with normal saline.
4. Check whether there are signs of infection such as redness, edema, bleeding, stench discharge of stoma when changing dress.
5. Disinfect the stoma area with 10% povidone–iodine from inside to outside area with 5 cm diameter and waiting 2 min to let it dry naturally.
6. Clean the 10% povidone–iodine with normal saline.
7. Record the healing, color, amount, and characteristics of secretion including result of wound culture.
8. Cover the clean stoma with aseptic Y gauze.
9. Change the ties of tracheal tube every 2 d or when it is contained.
10. Keep the tightness of the tie of tube around 1–2 fingers.

Dichotomous items (1 = yes, trainee do the step, 0 = no, trainee do not do the step); trainees will get 1 point whenever they do the correct step of stoma care for tracheostomy; maximal summarized *hands-on* care skill score is 10.

Before the end of the training, the preceptors provided more detailed information about care skills, such as raising the head of the bed before suction, opening a sterile suction catheter, inserting the catheter into the trachea without suction, applying suction intermittently while gently rotating the catheter and removing it, wrapping the disposable suction catheter around the dominant hand while withdrawing it, suctioning for no more than 10 seconds, providing oxygen after suction, assessing the airway, and repeating suction as necessary. During the discussion, descriptive feedback from trainees was recorded by the teaching assistance and included as part of the results.

### 2.5 Training protocols for the intervention group

In the *intervention* group, the 2-hour training began with a similar written test, self-assessed questionnaire on self-efficacy and satisfaction, and lecture as in the *regular* group, followed by an introduction to the topics and the purpose of the VR materials (30 minutes). Next, the preceptor demonstrated the first part of the HMD-VR material (15 minutes), and a volunteer trainee experienced the second part of the HMD-VR materials (15 minutes). During the preceptor demonstration and voluntary trainee experience with the HMD-VR materials, other trainees could simultaneously see the contents on the computer screen. Subsequently, every 2 trainees were subjected to a checklist-based audit of care skills for a tracheostomy stoma on manikins by an instructor who was blinded during the grouping of the trainees (30 minutes) (Table 4 and Fig. 2). Two preceptors were responsible for care skill auditing in this study. The preceptors responsible for auditing care skills were different from those of in-class preceptors. Finally, the preceptors led the open discussions and provided feedback (30 minutes), similar to that in the *regular* group. The trainees also completed written post-training tests and a self-assessed questionnaire on self-efficacy and satisfaction. Thus, every trainee became familiar with phone-based VR materials for use in clinical services (Fig. 2).

### 2.6 Sample size estimation

The primary and secondary endpoints were trainees’ satisfaction, trainees’ self-efficacy, and the degree of trainees’ application of web VR or text in educating patients’ families. We then estimated the number of trainees required to observe a 20% mean difference in the primary/secondary endpoints between the *regular* and *intervention* groups. The expected power was set at 80% and the type 1 error risk was set at 5%. For the mean difference (data with normal distribution) of primary/secondary endpoints, a 95% confidence interval indicated 2 standard deviations (SD) on either side of the mean difference (a total of 4 SD). Hence, 1 SD = 20/4 = 5, and the common variance (squared of SD) was 25. It was estimated that 30 patients in each of the *regular* and *intervention* groups (ie, 60 trainees in total) would be required for the evaluation.

### 2.7 Trainees

Between January 2018 and January 2020, healthcare providers involved in caring for patients who had undergone or were preparing for tracheostomy were invited to participate in this study. The healthcare providers volunteered to be trained (n = 67) and were invited to join our pilot text-based or VR material-based training to improve their ability to educate families about tracheostomy-related knowledge and care skills (Table 1). After excluding 7 healthcare providers due to incomplete training and questionnaires, a total of n = 60 individuals (28 physicians, 24 nurses and 8 respiratory therapists) were included in the final analysis of this study. Trainees were randomly divided into *regular* (14 physicians and 12 nurses vs 4 respiratory therapists) and *intervention* (14 physicians and 12 nurses vs 4 respiratory therapists) groups.

### 2.8 Self-efficacy and satisfaction with training and materials

Baseline demographic data (age, gender, discipline [*physician, nurse, or respiratory therapist*]), and the presence or absence of previous experience of training for tracheostomy-related knowledge and care skills were collected. At the baseline, post-training, and follow-up stages, each trainee assessed their self-efficacy (familiarity, confidence, and anxiety) for tracheostomy-related knowledge and care skills and satisfaction with the training and materials using the questionnaire list shown in Table 3.

### 2.9 Post-training implementation of education and service materials in clinical practice

After either *regular* or *intervention* VR training, trainees were asked to discuss the content of the text-based materials or smartphone-driven web-VR materials on tracheostomy-related knowledge and care skills with patients who had either undergone a tracheostomy or were preparing for and having a tracheostomy, and their families. For patients who had already undergone tracheostomy, information about daily care skills was introduced, whereas the content of the differences between ETT and tracheostomy was introduced to those preparing for tracheostomy and their families. The implementation of these materials in clinical practice was aimed at comforting patients and their families regarding care plans and procedures. Subsequently, trainees reported the degree of their patients’ or their families’ satisfaction with the clinical service and educational materials in follow-up questionnaires (Table 5).

**Table 5:** Questionnaires used at follow up stage after implementation of text-based or smartphone-based VR in clinical services.

For self-efficacy:
1. You are *familiar with* tracheostomy-related knowledge and care skills.
2. You have *confidence* in tracheostomy-related knowledge and care skills.
3. You are not *anxiety* about tracheostomy-related knowledge and care skills.
Satisfaction with the text-based or smartphone-based VR education and service materials:
1. These materials had increased the efficacy of my clinical services.
2. These materials had achieved the purposes of a paperless environment.
3. These materials had benefited my patients and families.
4. I had applied the learnt knowledge and skills from these materials on clinical practice.
5. I had recommended these materials to my patients’ families and patients who undergone or preparing for tracheostomy.
6. My patients and families agreed that these materials provided accurate messages about tracheostomy-related knowledge and care skills.
7. After using these materials, my patients and families are less anxious about tracheostomy procedure and care skills.

All questions are responded by the degree of agreement to the question [strongly agree (75%–100%), somewhat agree (50%–75%), not somewhat agree (25%–50%), not strongly agree (0%–25%), not applicable]. VR = virtual reality.

### 2.10 Follow-up assessments

Three to 4 weeks after training, follow-up questionnaires (Table 5) were administered to determine the sustained effects of training with either *regular* or *intervention* VR modules. The focus of self-evaluations included self-efficacy, trainees, and the satisfaction of their patients’ families with the educational materials about tracheostomy-related knowledge and care skills.

### 2.11 Ethics approval

Ethical approval (IRB No. 2019-12-007ACF) was granted by the Ethics Committee of the Taipei Veteran General Hospital. All voluntary trainees were informed about the importance and advantage of this intervention for their competencies, oral consent was obtained from all trainees, and questionnaire data were collected.

### 2.12 Statistical analysis

The comparison of the effects of either *regular* or *intervention* VR modules on self-efficacy on tracheostomy knowledge and care skills, satisfaction with the training, satisfaction with the text or VR materials, and senior preceptor-audited care skills scores were analyzed using *chi-square* tests for categorical variables or the Mann–Whitney test for continuous variables. Additionally, the corresponding data at the baseline, post-training, and follow-up stages were compared using one-way ANOVA. The comparison between the baseline and post-training data was analyzed using the Wilcoxon signed-rank test. All statistical analyses were performed using SPSS 21 (SPSS Inc., Chicago, IL), and statistical significance *was* set at *P* < .05.

### 2.13 Validity of the assessment tools

The content validity index (CVI) of the items of questionnaires evaluated by 2 experts, ranged from 0.67 to 0.9 (Table S1, Supplemental Digital Content). Item 10 of Table 2 shows the least value (0.67), meaning that it is considered less reliant than the others by the responder. In Table 1, the total summary content validity index is 0.83, indicating that experts acknowledged the excellent relevance of the scale for training objectives. In Tables S2 and S3, Supplemental Digital Content, the total summary content validity index of Tables 3 and 4 are 0.8 and 0.84, respectively, indicating that experts acknowledge the excellent relevance of the scale for training objectives.

## 3 Results

### 3.1 General characteristics

In this study, the case number, distribution of the proportion of disciplines, age range, mean age, and distribution of trainees with and without previous experience of training for tracheostomy-related knowledge and care skills did not differ between the *regular* and *intervention* groups (Table 1). Additionally, the degree of trainees*’* agreement (“strongly agree” or “somewhat agree”) to the statements of “*prior training met your need with respect to tracheostomy-related knowledge and care skills*”, “*prior training provided accurate messages about the tracheotomy-related knowledge and care skill*”, and “*you are happy to currently receive training on tracheostomy-related knowledge and care skills*” was not different between the *regular* and *intervention* groups.

### 3.2 VR intervention training increased trainees’ self-efficacy and their satisfaction with trainings and materials

**Figure 3:**
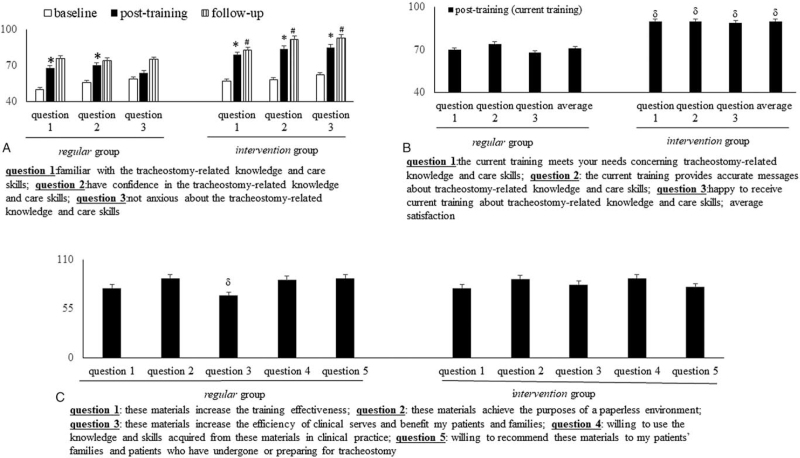
A comparison of the percentage of trainees who reported that they are “strongly agree” or “somewhat agree” with the individual statements in the questionnaire for evaluation of self-efficacy and satisfaction: (**A**) degree of trainees who reported that they “strongly agree” or “somewhat agree” with the statements related to self-efficacy [*familiarity*, *confidence*, *no anxiety*] with respect to tracheostomy -related knowledge and care skills; (**B**) degree of trainees who reported that they “strongly agree” or “somewhat agree” with the satisfaction-related statements on training; (**C**) degree of trainees who reported that they “strongly agree” or “somewhat agree” with the satisfaction-related statements about text or VR-based materials for tracheostomy-related knowledge and care skills. ^∗^*P* < .05 vs baseline data; ^#^*P* < .05 vs post-training data; ^δ^*P* < .05 vs data from the *regular* group. VR = virtual reality.

At the baseline stage, the degree of trainees’ agreement (“strongly agree” or “somewhat agree”) to the statements of “familiar with tracheostomy-related knowledge and care skills”, “have confidence in tracheostomy-related knowledge and care skills”, and “not anxious about tracheostomy-related knowledge and care skills” was not different between the intervention and regular groups (Fig. 3A). At the follow up stages, a higher percentage of intervention group trainees reported that they “strongly agree” or “somewhat agree” with the above-mentioned statements including familiarity (83 ± 4% vs 76 ± 2%, *P* = .04), confidence (92 ± 5% vs 74 ± 4%, *P* = .001) and reduced anxiety (93 ± 2% and 75 ± 6%, *P* = .002) compared to the regular group. Significantly, the VR intervention training module increased trainees’ familiarity, enhanced their confidence, and decreased their anxiety with respect to tracheostomy-related knowledge and care skills.

At the post-training stage, the average degree of trainees*’* agreement (“strongly agree” or “somewhat agree”) to the following statements “*the current training meets your needs concerning tracheostomy-related knowledge and care skills*”, “*the current training provides accurate messages about the tracheostomy-related knowledge and care skills*”, and “*happy to receive this training about the tracheostomy-related knowledge and care skills*” among the *intervention* group was higher (90 ± 6% vs 71 ± 2%, *P* = .007) than that in the *regular* group (Fig. 3B). At the follow-up stage, a higher degree (70 ± 2% vs 83 ± 8%, *P* = .005) of agreement (“strongly agree” or “somewhat agree”) to the statements of “*these materials increase the efficiency of clinical services and benefit your patients and families*” was observed among the *intervention* group’ trainees than *regular* group’ trainees (Fig. 3C). In addition to increasing self-efficacy, the new *intervention* model also improved trainees*’* satisfaction with training and materials.

### 3.3 Trainees reported that families of their patients were satisfied with VR educational materials-based clinical services

**Figure 4:**
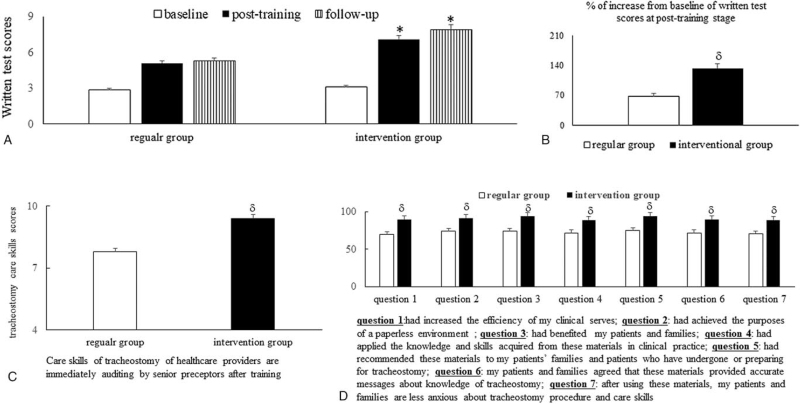
VR materials significantly increase knowledge, care skills, and trainee satisfaction. (**A**) Average of the trainees’ scores in the MCQ-based written test of tracheostomy-related knowledge and care skills (Table 1) at the baseline, post-training, and follow-up stages and (**B**) the degree of improvement in written test score from baseline; (**C**) average of the trainees’ scores in the hands-on skills test (Table 3) for tracheostomy care at the post-training stage; (**D**) the trainees reported degree of their patients/families “strongly agree” or “somewhat agree” with the questions on the feasibility of the text-based or VR materials for clinical services at the follow up stage. ^∗^*P* < .05 vs baseline data; ^δ^*P* < .05 vs data from the *regular* group. MCQ = multiple choice question, VR = virtual reality.

At the follow-up stage, the increasing trend of the average written test scores among the *intervention* group trainees was more significant (*P* = .006) than that in the *regular* group (Fig. 4A and B). After a direct audit by preceptors at the post-training stage, Fig. 4C shows the higher average hands-on tracheostomy care skills scores (9.4 ± 0.5 vs 7.8 ± 0.3, *P* = .002) in the *intervention* group*’* trainees compared to the *regular* group*’* trainees. The *intervention* group*’* trainees were more likely than the *regular* group*’* trainees to agree with statements on the feasibility of the materials for clinical services (Fig. 4D). These statements were as follows: **Statement 1**: *These materials have increased the efficiency of my clinical serves*; **Statement 2**: *These materials have achieved the purpose of a paperless environment*; **Statement 3**: *These materials have benefited my patients and families*; **Statement 4**: *I have applied the knowledge and skills acquired from these materials in clinical practice*; **Statement 5**: *I have recommended these materials to my patients and families who have preparing for tracheostomy and their families*; **Statement 6**: *My patients and their families agreed that these materials provided accurate messages*; **Statement 7**: *After using these materials, my patients and families are less anxious about the tracheostomy procedure and care skills* (Table 5).

### 3.4 Trainees gave positive feedbacks about VR trainings

In response to the open-ended questions posed at the end of our study, most trainees reported that this new *intervention* involving VR training significantly motivated them to improve tracheostomy-related knowledge and care skills. Specifically, the trainees reported that web VR materials are useful resources for refresher courses and self-directed learning.

A selection of completed feedback responses from the trainees are listed below.

1. This HMD-VR and web-VR training improved my knowledge of the indications for tracheostomy and of the differences between endotracheal intubation and tracheostomy with respect to feeding, suction, and speaking valve placement.
2. This training motivates me to introduce these self-accessible VR education and service materials to my patients and their families.
3. The process of introduction and hands-on practice of care skills in the VR world enhances my familiarity with tracheostomy-related knowledge and care skills and boosts my confidence in applying the acquired skills and knowledge in patient care.
4. I now understand the importance of knowledge-based communication with patients and their families in reducing their hesitation and improving quality of care.
5. Through this comprehensive training course, I understand that the main reasons for patients and their families to refuse tracheostomy include a loss of autonomy and poor self-image.
6. These newly developed VR materials provide accurate messages about the negative impacts of poor care skills-related complications.

## 4 Discussion

Good management of a patient with tracheostomy, both in the hospital and at home, has a significant impact on the quality of life of patients.^[34]^ Before the transition from inpatient to community care occurs, appropriate family education on care skills should be provided to avoid unnecessary and costly inpatient stays. In-hospital (nurses, physicians, and respiratory therapists) and community (family) healthcare providers are mainly responsible for taking care of patients with tracheostomies.^[35]^ A simulation-based tracheostomy educational program for in-hospital healthcare providers has been suggested to enhance the quality of care for these patients.^[27,36]^

In our study, we used HMD-VR and web-VR materials to educate in-hospital healthcare providers about tracheostomy care skills. Sophistication care skills may decrease the burden on families by reducing unnecessary hospital visits due to tracheostomy-related complications. Therefore, our trained in-hospital healthcare providers were asked to introduce these web VR materials to the patients’ families. Compared with text-or video-based training, an interactive VR setting can motivate training repetition, decrease the number of trainee errors, and increase the quantity of learning.^[37]^ To ensure the long-term competency of families’ tracheostomy care skills, our web-VR materials provide families with opportunities to refresh their knowledge of the content at home after an initial introduction by in-hospital healthcare providers.

Research has shown that 40% to 60% of patients could not correctly report what their healthcare providers expected of them 10 to 80 minutes after receiving medical information. Research has also demonstrated that over 60% of the patients interviewed immediately after consultation misunderstood the directions regarding prescribed medications.^[38]^ When it comes to complex medical knowledge, patients and their families, who have limited medical knowledge, recall as little as half of what was discussed during a typical medical encounter.^[39]^ Additionally, 1 study found that in 66% of the audiotaped cases analyzed, medical personnel omitted at least 1 piece of critical information when discussing new medical information with patients.^[40]^ When the medical encounter time is limited, medical personnel may fail to provide sufficient treatment-related knowledge to their patients and families.^[41]^ Additionally, communication without providing complete and accurate messages can negatively impact patient satisfaction and hamper adherence to care skills and outcomes.^[42]^

In our study, the degree of agreement with the statement “these materials increase the efficiency of clinical service and benefit your patients and families” was lower for the *regular* group trainees than for the *intervention* VR group trainees who used web-VR for clinical service. This clinically relevant difference may be due to the fact that the smartphone-driven web-VR materials provided families with the opportunity to refresh their tracheostomy-related knowledge and care skills at home repeatedly. The zoom-in and zoom-out approach, which can be accessed by scanning a QR code with a smartphone, is not limited by the capacity of full screen-displayed content such as text-based materials. Additionally, web-VR materials save human resources, space, and time required for setting up HMD-VR and increase the efficiency of clinical services.

VR technology enables more effective education at a lower cost and in less time than verbal or text-based methods by enhancing users’ incentive to engage.^[43]^ Predictably, by increasing accuracy messaging and decreasing the frequency of re-education of patients’ families, our new *intervention* training with smartphone-based VR materials is much less expensive than the *regular* training model.

A recent study reported that VR-based educational materials on chest radiography reduced children's anxiety and increased parents’ satisfaction with the procedure.^[44]^ Significantly, in addition to increasing self-efficacy with respect to tracheostomy-related knowledge and care skills, our newly developed VR educational materials decreased anxiety among trainees and families of their patients.

A few limitations of this study must be acknowledged. Our small sample size may have increased the risk of a type 2 error, limiting our ability to draw conclusions regarding the effect of the intervention. Our follow-up period was relatively short; we used a limited number of questions to investigate knowledge retention, and we provided answers to multiple-choice questions after trainees completed the baseline and post-training written tests. Although it was not easy for trainees to remember all the questions to the multiple-choice questions on the online questionnaire in the limited time available to them, the possibility that they used the answers given in the follow-up cannot be ruled out. In future studies, it will be important to use different pre- and post-test questionnaires to evaluate trainees’ attention and engagement. Additionally, the study could not be blinded due to the intervention property; it is very likely that the trainees were more satisfied with the novelty of the technique rather than the knowledge/skill they actually gained. To avoid bias in the subjective questionnaires, our study also included subjective written and direct auditing tracheostomy care skill tests to assess the outcomes.

In comparison with usual pain care among hospitalized patients, the adjuvant VR program for pain management was cost-saving as long as it reduced the length of hospital stay by ≥14.6%.^[45]^ For training of neonatal intensive care workers for hospital evacuation, the cost analysis revealed that VR-based disaster training is initially more expensive than live mannequin-based training in terms of the development costs. Nonetheless, when development costs are extrapolated to repeated training over 3 years, VR training becomes less expensive, while the cost of live training remains fixed. In other words, a larger initial investment in VR can be spread across a large number of trainees and a longer time period with little additional cost, whereas each live training requires additional costs that scale with the number of participants.^[46]^ In addition, a study examined ways to decrease the anxiety of pediatric patients before a chest film found,^[44]^ from the subjective feedback of patients, caregivers, and clinicians, that patients undergoing peripheral intravenous catheter placement who received VR intervention experienced significantly less anxiety than those who received standard care.^[47]^

Notably, our new VR *intervention* module achieved effects across levels 1 to 3 of the Kirkpatrick model.^[48]^ Further, the achievement of *Level 1 (reaction)* effects is indicated by the observation that trainees who underwent VR training were satisfied with the training and materials; the achievement of *Level 2 (learning)* is indicated by the observation that *interventional* training increased trainees’ tracheostomy-related knowledge and care skills (self-efficacy aspects such as familiarity and confidence); and the achievement of *Level 3 (behavior)* is indicated by the observation that the *intervention* group trainees applied the acquired knowledge and skills in clinical practice. The Kirkpatrick level 4 effects revealed in the current study include reports that trainees, their patients, and families agreed that these materials provided accurate messages and made them less anxious about the tracheostomy procedure and care skills. However, the limitations of the current study are the lack of an evaluation of the long-term effects of the VR program on the care levels as well as the cost effectiveness; hence, future studies need to compare whether using VR as a training modality is more cost-effective or reduces the repeated frequency of patient and family education for care skills to standard modules.

In conclusion, this study provides an example of the feasibility of using VR materials in clinical services. With the availability of self-accessible VR materials, medical trainees, patients, and families may have the opportunity to continuously refresh their corresponding knowledge and skills.

## Acknowledgments

The authors express their gratitude to all trainees for their input of this article.

## Author contributions

**Conceptualization:** Cheng-Hsien Wu, Chen-Huan Chen, Boaz Shulruf, Fa-Yauh Lee.

**Investigation:** Dung-Hung Chiang, Shu-Chuan Cheng, Ying-Ying Yang.

**Writing – original draft:** Jui-Chun Cheng, Ling-Yu Yang, Shou-Yen Kao.

**Writing – review & editing:** Chia-Chang Huang, Shiau-Shian Huang.

## Supplementary Material

Supplemental Digital Content

## Supplementary Material

Supplemental Digital Content

## Supplementary Material

Supplemental Digital Content
