# Supplementary material for: Immersive virtual reality (VR) training increases the self-efficacy of in-hospital healthcare providers and patient families regarding tracheostomy-related knowledge and care skills: A prospective pre–post study
Source: Medicine (Baltimore). 2022 Jan 14;101(2):e28570. doi: 10.1097/MD.0000000000028570 (PMC8757958; doi:10.1097/MD.0000000000028570)
Supplement: Supplemental Digital Content [file medi-101-e28570-s001.docx]

**Supplement Table 1. Content validity index evaluation by two experts for a checklist focused on auditing the care skills of trainees’ tracheostomy stoma**

| **Questions** | **ICVI-average** |
| --- | --- |
| 1. washing hands before caring of tracheostomy | 0.89 |
| 2. put on glove and remove Y gauze | 0.82 |
| 3. using cotton swab cleaning the stoma with normal saline | 0.76 |
| 4. check whether there are signs of infection such as redness, edema, bleeding, stench discharge of stoma when changing dress | 0.87 |
| 5. disinfect the stoma area with 10% povidone-iodine from inside to outside area with 5cm diameter and waiting 2 minutes to let it dry naturally | 0.8 |
| 6. clean the 10% povidone-iodine with normal saline | 0.9 |
| 7. record the healing, color, amount and characteristics of secretion including result of wound culture | 0.84 |
| 8. cover the clean stoma with aseptic Y gauze | 0.83 |
| 9. change the ties of tracheal tube every two days or when it is contained | 0.9 |
| 10. keep the tightness of the tie of tube around 1-2 fingers | 0.67 |
|  | Average S-CVI=0.83 |
