# Supplementary material for: Immersive virtual reality (VR) training increases the self-efficacy of in-hospital healthcare providers and patient families regarding tracheostomy-related knowledge and care skills: A prospective pre–post study
Source: Medicine (Baltimore). 2022 Jan 14;101(2):e28570. doi: 10.1097/MD.0000000000028570 (PMC8757958; doi:10.1097/MD.0000000000028570)
Supplement: Supplemental Digital Content [file medi-101-e28570-s002.docx]

**Supplement Table 2 The CVI was evaluated by two experts in a questionnaire for trainees’ self-assessed self-efficacy and satisfaction**

| **Questions** | **ICVI-average** |
| --- | --- |
| **Baseline** self-efficacy: |  |
| 1.You are *familiar with* the knowledge and care skills of tracheostomy | 0.9 |
| 2.You have *confidence* in the knowledge and care skills of tracheostomy | 0.85 |
| 3.You are not *anxiety* about the knowledge and care skills of tracheostomy | 0.8 |
| **Baseline** satisfaction to the prior training: |  |
| 1.Prior training that you had already met your need on the knowledge and care skills of tracheotomy | 0.75 |
| 2.Prior training that you had already provided accurate messages about the knowledge and care skills of tracheotomy | 0.8 |
| 3.You are happy to receive current training about the knowledge and care skills of tracheostomy | 0.85 |
| **Post-training** self-efficacy: |  |
| 1.you are *familiar with* the knowledge and care skills of tracheostomy | 0.85 |
| 2.you have *confidence* in the knowledge and care skills of tracheostomy | 0.75 |
| 3.you are not *anxiety* about the knowledge and care skills of tracheostomy | 0.95 |
| **Post-training** satisfaction to the current training: |  |
| 1. the current training meet your needs on the knowledge and care skills of tracheostomy | 0.75 |
| 2.the current training provides accurate messages about the knowledge and care skills of tracheostomy | 0.85 |
| 3.you are happy to receive current training about the knowledge and care skills of tracheostomy | 0.75 |
| **Post-training** satisfaction to text-based or smartphone-based VR training and service materials |  |
| 1. these materials increase the effectiveness of training | 0.85 |
| 1. these materials achieve the purpose of paperless | 0.9 |
| 1. these materials will increase the efficacy of clinical serves and benefit your patients and families | 0.8 |
| 1. you are willing to use the learnt knowledge and skills from these materials on clinical practice | 0.95 |
| 1. you are willing to recommend these materials to my patients’ families and patients whose are preparing for and having tracheostomy | 0.85 |
|  | Average S-CVI=0.84 |
