# Supplementary material for: Immersive virtual reality (VR) training increases the self-efficacy of in-hospital healthcare providers and patient families regarding tracheostomy-related knowledge and care skills: A prospective pre–post study
Source: Medicine (Baltimore). 2022 Jan 14;101(2):e28570. doi: 10.1097/MD.0000000000028570 (PMC8757958; doi:10.1097/MD.0000000000028570)
Supplement: Supplemental Digital Content [file medi-101-e28570-s003.docx]

**Supplement Table 3 CVI evaluation by two experts for questionnaires used at the follow-up stage after the implementation of text-based or smartphone-based VR in clinical services**

| **Questions** | | **ICVI-average** |
| --- | --- | --- |
| **For self-efficacy**: | |  |
| 1. you are *familiar with* the knowledge and care skills of tracheostomy | 0.9 |  |
| 2. you have *confidence* in the knowledge and care skills of tracheostomy | 0.8 |  |
| 3. you are not *anxiety* about the knowledge and care skills of tracheostomy: | 0.8 |  |
| **Satisfaction to the text-based or smartphone-based VR education and service materials**: | |  |
| 4.these materials had increased the efficacy of my clinical services | | 0.9 |
| 5.these materials had achieved the purpose of paperless | | 0.8 |
| 6.these materials had benefited my patients and families | | 0.85 |
| 7.I had applied the learnt knowledge and skills from these materials on clinical practice | | 0.75 |
| 8.I had recommended these materials to my patients’ families and patients whose are preparing for or having tracheostomy | | 0.75 |
| 9.my patients and families agreed that these materials provided accurate messages about the knowledge and care skills of tracheostomy | | 0.7 |
| 10.after using these materials, my patients and families are less anxiety about the procedure and care skills of tracheostomy” | | 0.75 |
|  | | Average S-CVI=0.8 |
